# Assessing the Significance and Awareness of Oral Collagen in Enhancing Health and Beauty Among Consumers in Saudi Arabia: A Cross-Sectional Study

**DOI:** 10.7759/cureus.91149

**Published:** 2025-08-27

**Authors:** Hind Khalid Goresh, Sultan H Almarwani, Ftoon Alhomidani, Faisal A Alharbi, Jayiz S Alharbi, Noura Alkatheri, Nourah M Alamro, Mabrouk Al-Rasheedi, Balqees Alkharisi, Rahaf Alwakr

**Affiliations:** 1 Department of Clinical Pharmacy, Buraydah Private Colleges, Buraydah, SAU; 2 Department of Pharmacology, Buraydah Private Colleges, Buraydah, SAU; 3 College of Pharmacy and Dentistry, Buraydah Private Colleges, Buraydah, SAU; 4 Department of Clinical Research, Al-Bukayriyah General Hospital, Bukayriyah, SAU

**Keywords:** collagen supplements, consumer awareness, joint pain, quality of life, skin health

## Abstract

Background

Oral collagen supplements have become increasingly popular in Saudi Arabia, as they are widely recognized for their significant benefits to skin health, joint support, and overall wellness. This study aimed to evaluate the significance of oral marine collagen in enhancing health and beauty among Saudi consumers.

Method

A cross-sectional survey involving 171 participants was conducted to evaluate the effectiveness, usage, side effects, and reasons for discontinuation of the product. Data were analyzed using descriptive statistics, Chi-square tests, and a logistic regression model using SPSS version 29.0.0 (IBM Corp., Armonk, NY).

Results

Among 171 participants, the majority were women (153, 89.5%) and aged 20-30 years (78, 45.6%). Collagen use was reported by 56 (32.7%), mainly for strengthening hair/nails (42, 75%) and improving skin (40, 71.4%). Reported improvements included hair/nails (13, 23.2%), with 25 (44.6%) experiencing some improvement; skin (13, 23.2%), with 24 (42.9%) experiencing some improvement; joint pain (12, 21.4%), with 22 (39.3%) experiencing some improvement. Powder (45, 80.4%) was the most used form. Users of collagen for 4-8 weeks had significantly greater hair/nail improvement (73.7% versus 26.3%, p=0.033) and lower early discontinuation (16.7% versus 50.0%, p=0.010). Side effects were reported by 32 (57.1%), with gastrointestinal (GIT) symptoms in 18 (32.1%). Sleep duration improved post-supplementation, but not significantly (p=0.175). Age was the only significant predictor of collagen benefit (p=0.002), with younger participants more likely to report positive outcomes.

Conclusions

This study shows that oral collagen use is associated with modest improvements in hair, skin, joint comfort, and sleep, particularly with consistent use over 4-8 weeks. Younger individuals experienced greater benefits. However, side effects and early discontinuation were common. Improved public awareness and further research are recommended to optimize safe and effective use.

## Introduction

Collagen is an essential protein that serves as a key structural component of the skin, helping to maintain its elasticity, firmness, and hydration. This protein not only supports the skin's architecture but also plays a significant role in enhancing the strength and resilience of hair and nails. In the context of bones and teeth, type I collagen constitutes the majority of the organic matrix, providing the necessary resistance and rigidity that protects against fractures and injuries. It acts as a framework that allows these structures to withstand mechanical stress and maintain their integrity [1].

Furthermore, collagen is vital in the composition of tendons and ligaments, where it facilitates the transmission of forces between muscles and bones. This function is crucial for movement and stability, allowing athletes and active individuals to perform physical activities efficiently. Additionally, collagen has the ability to store elastic energy, which contributes to the flexibility and agility of the musculoskeletal system [2]. Type II collagen, on the other hand, is predominantly found in articular cartilage, which cushions joints and enables smooth motion. This type of collagen is essential for maintaining joint health and preventing conditions such as osteoarthritis.

Despite its numerous benefits, collagen supplementation is not without potential side effects. These may include an increased risk of developing kidney stones, liver abnormalities, gastrointestinal issues, excessive calcium buildup in the body, and symptoms such as headaches, dizziness, and allergic reactions in some individuals. It is important for those considering collagen supplements to consult with a healthcare professional to ensure safety and appropriateness for their specific health needs [3].

## Materials and methods

This study employs a cross-sectional survey design, utilizing a convenience sample of individuals aged 18 years and older. Participants under the age of 18 were excluded from the study. The questionnaire was developed using Google Forms (Google, Inc., Mountain View, CA) and consisted of two main sections: one focused on collecting socioeconomic data and the other on assessing the effectiveness of collagen, knowledge about the products, side effects, usage pattern, duration of use, and adherence. The questionnaire included both open- and closed-ended questions to gather a range of opinions, without focusing on identifying right or wrong answers. Additionally, a market survey was conducted to analyze collagen-based products available both online and in stores. The survey questionnaire was tested with 20 individuals prior to being distributed on social media platforms (Instagram, Snapchat, and WhatsApp) in groups likely to use this type of supplement, including athletes, individuals with musculoskeletal disorders, and those with skin issues, among others. These questions were developed based on information gathered from literature and advertisements for collagen products in Saudi Arabia, as such information is more readily available to the public. The inquiries regarding knowledge and attitude focused on the motivations for consuming collagen and the duration of its use. Finally, the investigation into significance and practice involved questions about participants' views on the benefits or improvements to their skin and overall health resulting from collagen consumption. An exploratory methodology was employed to gather data on the characteristics of consumers of collagen-based products, using a validated survey instrument that integrates questions assessing knowledge along with attitudinal and significance scales to evaluate both constructs scientifically. Data analysis was carried out using Microsoft Excel 2013 (Microsoft Corp., Redmond, WA) and SPSS version 29.0.0 (IBM Corp., Armonk, NY). Descriptive statistics were applied to present frequencies, percentages, and means, while the Chi-square (χ²) test was utilized to examine associations between knowledge, attitudes, and practices (KAP) regarding collagen and various sociodemographic variables.

## Results

A total of 171 participants were included, with the majority being women (153, 89.5%), and only 18 (10.5%) were men (Table 1). Most were aged 20-30 years (78, 45.6%), followed by 40-50 years (38, 22.2%), 30-40 years (21, 12.3%), 18-20 years (19, 11.1%), and >50 years (15, 8.8%). Regarding weight, 50-60 kg was the most common (47, 27.5%), followed by 60-70 kg (45, 26.3%), 70-80 kg (30, 17.5%), <50 kg (17, 9.9%), 80-90 kg (20, 11.7%), and >90 kg (12, 7%). Educationally, most held a bachelor's degree (116, 67.8%), with fewer holding a diploma (26, 15.2%), secondary education or less (20, 11.7%), or postgraduate degrees (9, 5.3%). Monthly income was >5,000 SAR in 76 (44.4%) and <3,000 SAR in 72 (42.1%). The majority resided in the Central Region (136, 79.5%). Collagen use was reported by 56 (32.7%), while 115 (67.3%) had never used it, with the most common reasons being lack of need (55, 47.8%), followed by doubts about effectiveness (25, 21.7%), fear of side effects (21, 18.3%), and high price (14, 12.2%).

**Table 1:** Sociodemographic characteristics and other details of the participants (N=171)

Sociodemographic characteristics and other details		Frequency (number (%))
Gender	Female	153 (89.5%)
	Male	18 (10.5%)
Age	<20 years	19 (11.1%)
	20-30 years	78 (45.6%)
	30-40 years	21 (12.3%)
	40-50 years	38 (22.2%)
	>50 years	15 (8.8%)
Weight	<50 kg	17 (9.9%)
	50-60 kg	47 (27.5%)
	60-70 kg	45 (26.3%)
	70-80 kg	30 (17.5%)
	80-90 kg	20 (11.7%)
	>90 kg	12 (7%)
Educational level	Secondary or lower	20 (11.7%)
	Diploma	26 (15.2%)
	Bachelor's	116 (67.8%)
	Postgraduate	9 (5.3%)
Monthly income level	<3,000 SAR	72 (42.1%)
	3,000-4,000 SAR	18 (10.5%)
	4,000-5,000 SAR	5 (2.9%)
	>5,000 SAR	76 (44.4%)
Area	Central Region	136 (79.5%)
	Western Region	19 (11.1%)
	Northern Region	8 (4.7%)
	Southern Region	4 (2.3%)
	Eastern Region	4 (2.3%)
Ever used collagen supplements?	No	115 (67.3%)
	Yes	56 (32.7%)
Reasons for not using collagen	Not needed	55 (47.8%)
	Lack of confidence in the effectiveness	25 (21.7%)
	Fear of side effects	21 (18.3%)
	High price	14 (12.2%)

**Figure 1:**
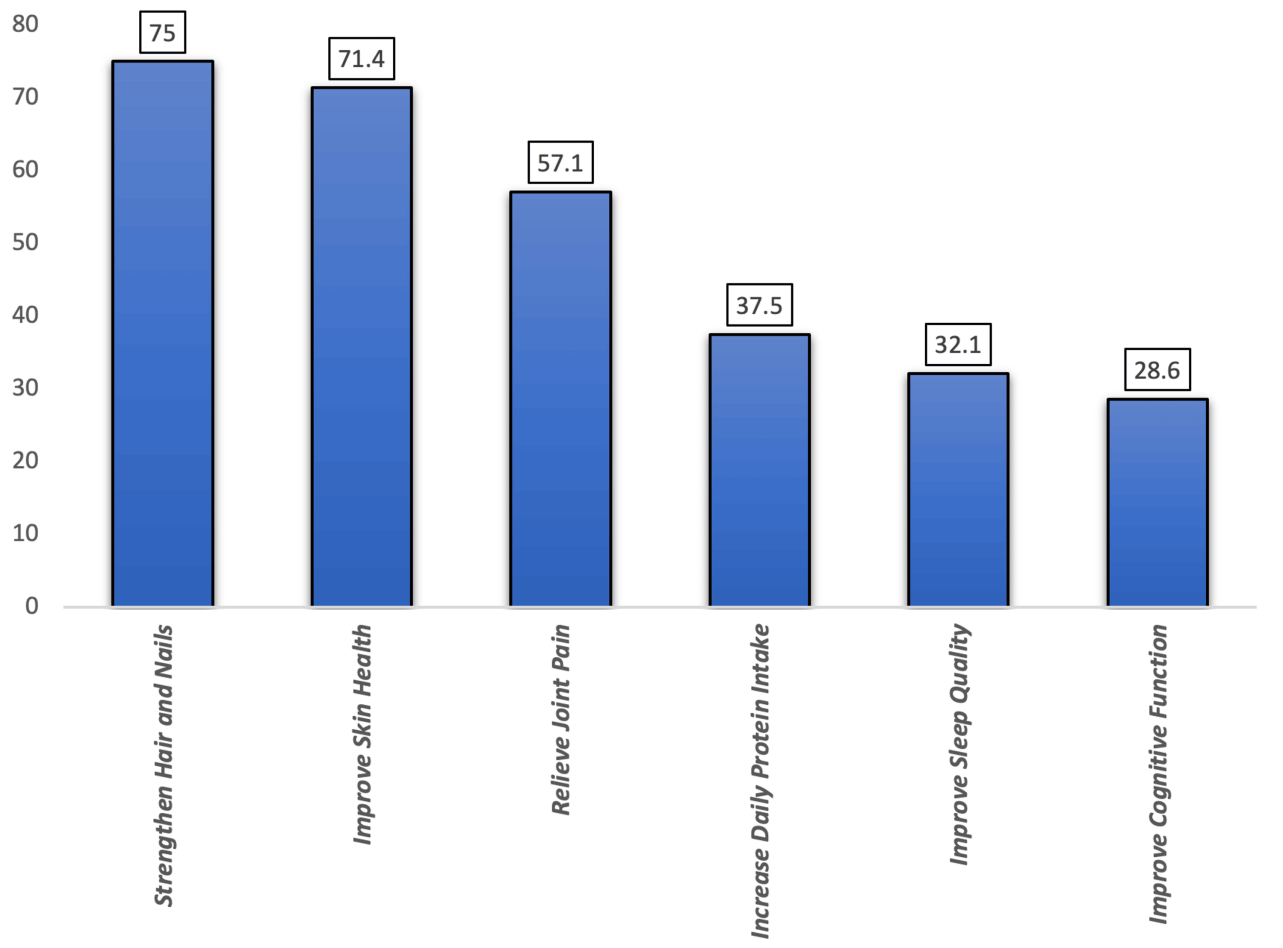
Reasons for using collagen among the participants (n=56)

Figure 1 shows the reasons for collagen use among participants (n=56). The most frequently reported reason was to strengthen hair and nails in 42 (75%) participants, which is followed by improving skin health in 40 (71.4%). A total of 32 (57.1%) used collagen to relieve joint pain, while 21 (37.5%) reported using it to increase daily protein intake. Additionally, 18 (32.1%) took collagen to improve sleep quality, and 16 (28.6%) aimed to enhance cognitive function.

Table 2 shows the impact of collagen on several quality of life (QoL) parameters. Hair/nail improvement was noted by 13 (23.2%), while 25 (44.6%) experienced some improvement. Similarly, skin enhancement was reported by 13 (23.2%), and some improvement by 24 (42.9%). Joint pain improved in 12 (21.4%), with partial relief in 22 (39.3%). For protein intake, 10 (17.9%) reported improvement, while 18 (32.1%) experienced some benefit. Sleep quality improved in 11 (19.6%) and somewhat in 21 (37.5%). Regarding the dosage and formulation, powder (45, 80.4%) and capsules (37, 66.1%) were the most used forms. The most common dosage was 2.5-5 gm (10, 17.9%). Most of the participants used collagen for 4-6 weeks (21, 37.5%). Most of them were aware that the side effects of collagen included gastrointestinal (GIT) disorders (21, 37.5%). Notably, 32 (57.1%) experienced symptoms, with 18 (32.1%) reporting GIT-related issues and 17 (30.4%) discontinuing collagen due to side effects. Over half (57.1%) discontinued collagen prematurely before the expected results.

**Table 2:** Impact of collagen use after one month on the QoL of patients (n=56) GIT: gastrointestinal, QoL: quality of life

Parameters		Frequency (number (%))
Hair/nail improvement	No	18 (32.1%)
	Somewhat	25 (44.6%)
	Yes	13 (23.2%)
Skin improvement	No	19 (33.9%)
	Somewhat	24 (42.9%)
	Yes	13 (23.2%)
Joint pain improvement	No	22 (39.3%)
	Somewhat	22 (39.3%)
	Yes	12 (21.4%)
Protein intake improvement	No	28 (50.0%)
	Somewhat	18 (32.1%)
	Yes	10 (17.9%)
Sleep quality improvement	No	24 (42.9%)
	Somewhat	21 (37.5%)
	Yes	11 (19.6%)
Sleep hours before collagen	<4 hours	7 (12.5%)
	4-6 hours	26 (46.4%)
	6-8 hours	18 (32.1%)
	>8 hours	5 (8.9%)
Sleep hours after collagen	<4 hours	3 (5.4%)
	4-6 hours	19 (33.9%)
	6-8 hours	28 (50.0%)
	>8 hours	6 (10.7%)
Parameters of collagen usage		Frequency (number (%))
Daily dosage of collagen	2.5 gm	7 (12.5%)
	2.5-5 gm	10 (17.9%)
	5-10 gm	8 (14.3%)
	10-15 gm	4 (7.1%)
	>15 gm	4 (7.1%)
	Other	17 (30.4%)
Awareness of available forms	Capsules	46 (82.1%)
	Gummy	32 (57.1%)
	Needles	10 (17.9%)
	Powder	48 (85.7%)
Form used by participants	Capsules	37 (66.1%)
	Gummy candy	28 (50.0%)
	Needles	8 (14.3%)
	Powder	45 (80.4%)
Duration of collagen use	<4 weeks	20 (35.7%)
	4-6 weeks	21 (37.5%)
	6-8 weeks	6 (10.7%)
	>8 weeks	9 (16.1%)
Awareness about the side effects of collagen	GIT disorders	21 (37.5%)
	Kidney stones	9 (16.1%)
	Headache	3 (5.4%)
	Liver disease	4 (7.1%)
	Other symptoms	19 (33.9%)
Experienced symptoms from collagen	GIT disorders	18 (32.1%)
	Kidney stones	1 (1.8%)
	Headache	5 (8.9%)
	Other Symptoms	32 (57.1%)
Appearance of side effects may cause discontinuation of the drug	No	24 (42.9%)
	Don't know	15 (26.8%)
	Yes	17 (30.4%)
Did you stop taking collagen before the expected result?	No	24 (42.9%)
	Yes	32 (57.1%)

**Figure 2:**
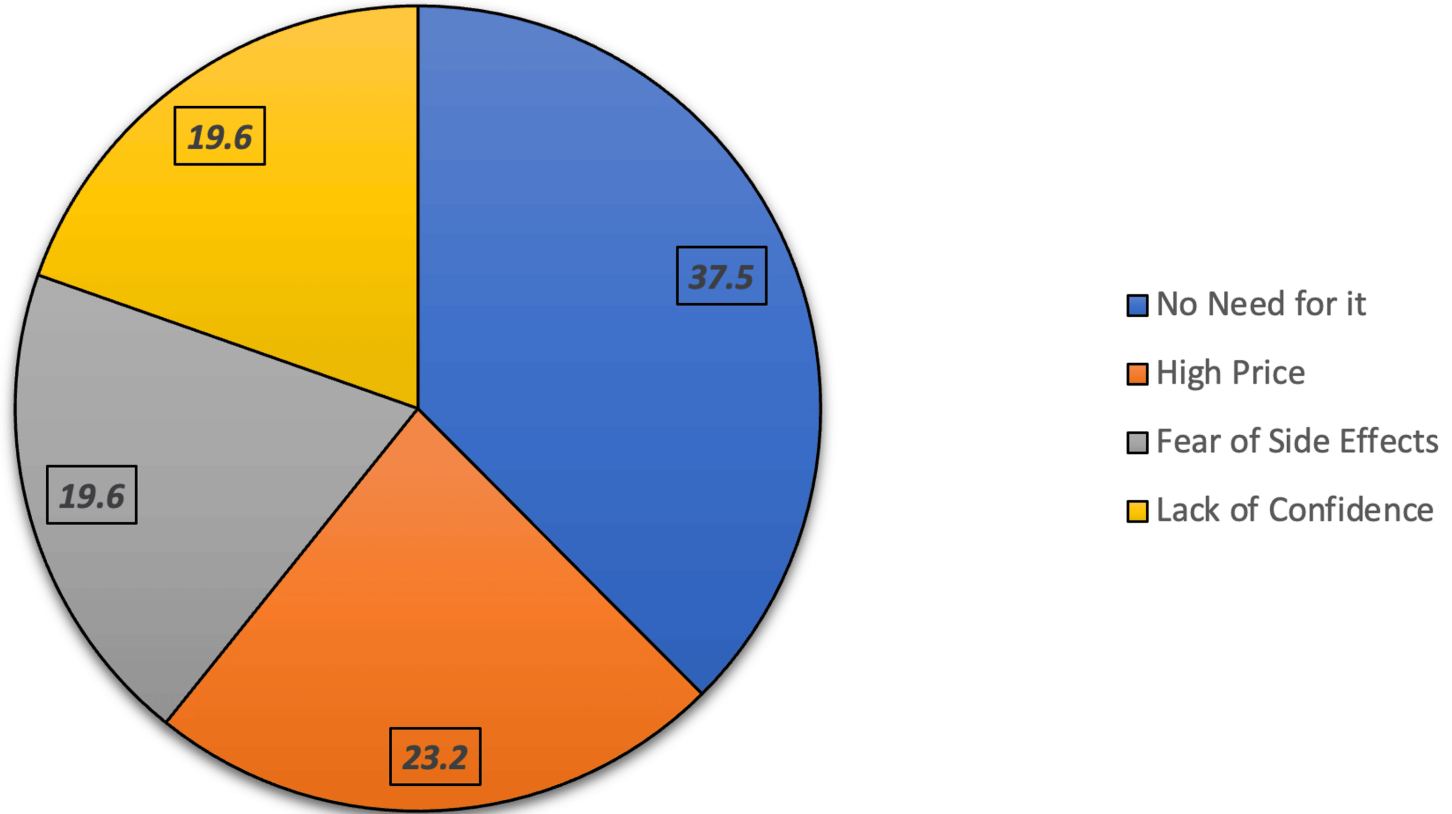
Reason for stopping collagen before the expected results (n=32)

Figure 2 shows the reasons for discontinuing collagen use before the expected results (n=32). The most commonly cited reason was no longer feeling the need for it, reported by 12 (37.5%) participants. This was followed by high price in seven (23.2%), while fear of side effects and lack of confidence in effectiveness were each reported by six (19.6%) participants.

**Figure 3:**
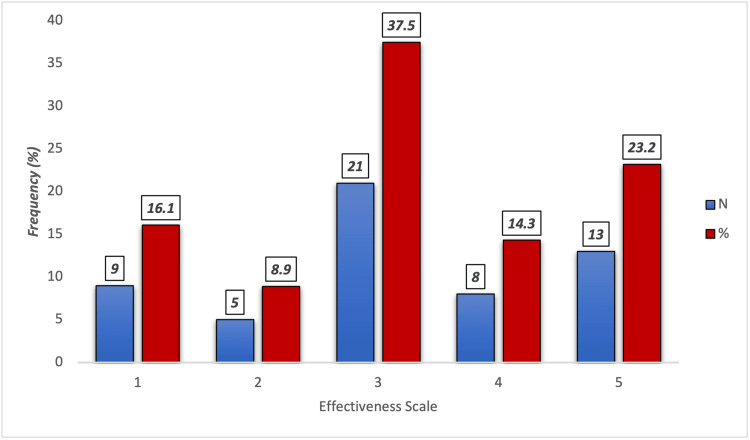
Perceived effectiveness of collagen in improving joint and bone pain (0: no effectiveness, 5: highest effectiveness) (n=32)

Figure 3 presents the perceived effectiveness of collagen in improving joint and bone pain among participants (n=32), rated on a scale from 0 (no effectiveness) to 5 (highest effectiveness). The most common rating was 3, reported by 12 (37.5%) participants, indicating a moderate perceived benefit. Thirteen (23.2%) participants rated it at the highest level (5), and eight (14.3%) gave it a 4. Lower effectiveness ratings were given by nine (16.1%) participants at level 1 and five (8.9%) at level 2.

Table 3 shows the participants' awareness regarding the impact of collagen use on quality of life. The mean overall awareness score was 6.94 (standard deviation (SD): 4.32), with a median of 7.00, which indicates that there is a generally low-to-moderate level of knowledge. The participants showed the highest awareness in understanding the reasons for collagen use, with a mean score of 1.68 (SD: 1.00) and a median of 2.00 (range: 0-4). Awareness regarding expected results after continued use followed with a mean of 1.35 (SD: 1.11). Knowledge about the side effects of collagens had a mean score of 1.08 (SD: 1.09), while the understanding about the period of its use scored slightly lower at 0.98 (SD: 1.02). Familiarity with the available pharmaceutical forms of collagen averaged 0.97 (SD: 0.92), and awareness of the collagen's benefits in improving sleep quality was the lowest, with a mean score of 0.88 (SD: 0.86).

**Table 3:** Awareness about the impact of collagen outcome on the QoL (higher score shows higher awareness) SD: standard deviation, min: minimum, max: maximum, QoL: quality of life

	Mean (SD)	Median	Range (min-max)
Extent of knowledge and awareness of the reasons for use	1.68 (1.00)	2.00	0-4
Awareness and knowledge of the side effects associated with taking collagen	1.08 (1.09)	1.00	0-4
Extent of awareness and knowledge of the period of use according to each case	0.98 (1.02)	1.00	0-4
Extent of knowledge and awareness of the expected results after continued use	1.35 (1.11)	2.00	0-4
Knowledge about the benefits of collagen in improving sleep quality	0.88 (0.86)	1.00	0-4
Familiarity with the available pharmaceutical forms of collagen	0.97 (0.92)	1.00	0-4
Overall awareness about the use and outcome of collagen use	6.94 (4.32)	7.00	0-17

Table 4 shows the association between the duration of collagen usage and its impact on various quality of life parameters. Participants who used collagen for 4-8 weeks showed significantly greater hair/nail improvement compared to those using it for <4 weeks (73.7% versus 26.3%, p=0.033). While skin and joint pain improvements were more frequent in those who used collagen for 4-8 weeks (73.0% and 73.5%, respectively), these parameters did not reach statistical significance (p=0.058 and p=0.073, respectively). Improvements in protein intake and sleep quality were also more commonly reported in the 4-8 weeks group, but differences were not statistically significant (p>0.05). Among the reported side effects, GIT disorders were more common in the 4-8 weeks group (72.2%), but this was not statistically significant (p=0.308). Notably, premature discontinuation of collagen was significantly lower in the 4-8 weeks group (16.7%) as compared to the <4 weeks group (50%), with a p-value of 0.010.

**Table 4:** Association between duration of collagen usage and its impact on QoL ^a^Chi-square test ^b^Fisher's exact test p<0.05: statistically significant QoL: quality of life, GIT: gastrointestinal

Parameters		<4 weeks (number (%))	4-8 weeks (number (%))	Chi-square values	Significant values
Hair/nail improvement	No	10 (55.6%)	8 (44.4%)	4.54	0.033^a^
	Yes	10 (26.3%)	28 (73.7%)		
Skin improvement	No	10 (52.6%)	9 (47.4%)	3.58	0.058^a^
	Yes	10 (27.0%)	27 (73.0%)		
Joint pain improvement	No	11 (50.0%)	11 (50.0%)	3.22	0.073^a^
	Yes	9 (26.5%)	25 (73.5%)		
Protein intake improvement	No	12 (42.9%)	16 (57.1%)	1.24	0.265^a^
	Yes	8 (28.6%)	20 (71.4%)		
Sleep quality improvement	No	10 (41.7%)	14 (58.3%)	0.64	0.421^a^
	Yes	10 (31.3%)	22 (68.8%)		
Symptoms experienced	GIT disorders	5 (27.8%)	13 (72.2%)	0.42	0.308^b^
	Kidney stones	1 (100.0%)	0 (0.0%)		
	Headache	3 (60.0%)	2 (40.0%)		
	Other symptoms	11 (34.4%)	21 (65.6%)		
Stopped collagen prematurely before the effect appears	No	4 (16.7%)	20 (83.3%)	6.63	0.010^a^
	Yes	16 (50.0%)	16 (50.0%)		

Table 5 shows the positive impact of collagen use over a two-month period on sleep duration and QoL. After collagen supplementation, an upward trend in sleep hours was observed, although the difference was not statistically significant (p=0.175). The proportion of participants sleeping <4 hours decreased from seven (70%) before to three (30%) after collagen use. Likewise, those sleeping 4-6 hours declined from 26 (57.8%) to 19 (42.2%). In contrast, the number of participants reporting 6-8 hours of sleep increased from 18 (39.1%) to 28 (60.9%), and those sleeping >8 hours rose from five (45.5%) to six (54.5%).

**Table 5:** Impact of using collagen for two months on the duration and quality of sleep ^a^Fisher's exact test, p<0.05: statistically significant

Parameter		Before collagen (number (%))	After collagen (number (%))	Fisher-Freeman-Halton exact test	^a^Significant value
Sleep fours	<4 hours	7 (70.0%)	3 (30.0%)	4.89	0.175
	4-6 hours	26 (57.8%)	19 (42.2%)		
	6-8 hours	18 (39.1%)	28 (60.9%)		
	>8 hours	5 (45.5%)	6 (54.5%)		

Table 6 shows the adjusted predictors of the good impact of collagen on quality of life (QoL) among the participants. Age was the only statistically significant predictor (p=0.002), where younger participants were more likely to experience a positive impact from collagen use (B=-0.610, odds ratio (OR)=0.543, 95% confidence interval (CI): 0.366-0.805). Although they are not statistically significant, higher education level (B=-0.427, p=0.062, OR=0.652) and higher monthly income (B=0.237, p=0.105, OR=1.268) showed near-significant trends, which suggest that there is a possible influence of these parameters on QoL of collagen users. Gender (B=-0.982, p=0.110) and weight (B=0.076, p=0.578) were not significant predictors.

**Table 6:** Adjusted predictors of the good impact of collagen on QoL among the participants B: Regression coefficient, Sig: significance level (p<0.05), Exp (B): odds ratio, CI: confidence interval

Predictors	B	Sig.	Exp(B)	95% CI for Exp(B)
Gender (male)	-0.982	0.110	0.374	0.112-1.250
Age (years)	-0.610	0.002	0.543	0.366-0.805
Weight (kg)	0.076	0.578	1.079	0.825-1.413
Higher educational level	-0.427	0.062	0.652	0.417-1.021
Higher monthly income	0.237	0.105	1.268	0.952-1.688
Constant	1.116	0.103	3.052	-

## Discussion

Collagen is a structural protein that is very important for skin elasticity, firmness, and hydration. It also helps in strengthening hair and nails [4]. In bones and teeth, type I collagen constitutes the organic matrix, which provides resistance to fractures. It supports the tendons and ligaments by force transmission and enables movement, stability, and elastic energy storage [5]. Type II collagen predominates in the articular cartilage, which provides cushions to joints and prevents osteoarthritis [6]. Although collagen is safe, it may lead to adverse effects, such as kidney stones, liver abnormalities, gastrointestinal issues, calcium accumulation, headaches, dizziness, or allergic reactions [7]. Consultation with a healthcare professional is advised to ensure safety and appropriateness. This study aims to evaluate the significance of oral marine collagen in enhancing health and beauty among Saudi consumers.

Notably, this study shows that only 32.7% of the participants had previously used collagen, despite good awareness about its availability. A systematic review by Shenoy et al. (2022) shows that the increasing rate reflects the emerging trend in wellness and nutraceutical sectors across the Gulf Region, where marine collagen has gained popularity for its benefits for skin health, joint support, and anti-aging effect [8]. Moreover, the primary reasons for the use of collagen were to strengthen hair and nails (75%) and improve skin health (71.4%), followed by relieving joint pain (57.1%). These motivating factors are consistent with global consumer behavior and aligned with the existing clinical literature by Geahchan et al. (2022), which showed that marine-derived hydrolyzed collagen supports dermal integrity and joint cartilage regeneration over prolonged periods of intake [9].

One of the important findings of our study is that the participants who used collagen for at least one month have reported varying degrees of improvement across multiple QoL domains. Specifically, 23.2% noted full improvement in hair and nail health, and 44.6% reported partial improvement. Furthermore, similar trends were seen for skin enhancement, where 23.2% observed noticeable changes, and 42.9% reported modest gains. These findings aligned with the results from previous literature by Pu et al. (2023), which showed that there is an improvement in skin elasticity, hydration, and roughness with daily oral collagen, especially after 8-12 weeks of consistent intake [10]. While our study only observed these effects at a short-term duration of two months, their benefits would manifest with longer-term use, as supported by prior research.

In terms of joint pain, 21.4% of the users reported full relief, while 39.3% experienced partial improvement. These self-reported outcomes are noteworthy based on the short duration of collagen use in most of the participants, and these are consistent with clinical trials that showed collagen's role in reducing joint discomfort, especially in osteoarthritis and aging populations. Similarly, Martínez-Puig et al. (2023) show that the native type II collagen has reported positive results in terms of pain relief and joint function improvement [11]. The reported perception of moderate effectiveness (score 3 out of 5) by 37.5% in our participants further supports its potential in enhancing musculoskeletal comfort.

Interestingly, our study also explored the outcomes that are not frequently discussed in the previous literature, such as sleep quality and protein intake. Improvements in sleep were reported by 19.6% of users, and 32.1% reported some improvement in sleep. While evidence-based literature on collagen's impact on sleep is limited, the findings of our study suggested that there may be a possible indirect effect, which is due to better joint comfort or gut health. The study by Thomas et al. (2024) shows that collagen peptide supplementation did not influence sleep quantity, latency, or efficiency, but reduced awakenings and improved cognitive function in physically active men with sleep complaints [12]. Similarly, the reported increase in daily protein intake among 17.9% of users reflects collagen's contribution as a supplemental protein source, which may benefit individuals with low dietary protein.

According to our findings, the duration of collagen use had a clear relationship with the outcomes. The participants who used collagen for 4-8 weeks showed that there were significantly better outcomes in hair and nail improvement (p=0.033), and they were less likely to discontinue the usage prematurely (16.7% versus 50.0%, p=0.010). These findings reinforce the importance of consistent, prolonged supplementation to observe noticeable results, as suggested in a meta-analysis by Pu et al. (2023), which highlighted at least 12 weeks of daily intake for maximum effect [10].

Despite these encouraging findings, side effects and discontinuation were common. Gastrointestinal (GIT) symptoms were reported by 32.1% of users, making them the most frequent adverse effect. A total of 57.1% experienced some side effects, and 30.4% discontinued collagen due to these symptoms. A study by Al Bahri et al. (2022) shows that collagen supplements can trigger adverse cutaneous reactions ranging from generalized bullous fixed drug eruption, maculopapular rash, drug reaction with eosinophilia and systemic symptoms (DRESS) syndrome, and bullous erythema multiforme to severe Stevens-Johnson syndrome/toxic epidermal necrolysis, as well as immediate hypersensitivity responses [13].

Moreover, awareness regarding collagen uses and its outcomes was suboptimal. The mean awareness score was 6.94 out of a possible 17, with some notable gaps in understanding the correct duration of use, expected outcomes, and potential side effects. The multivariate regression analysis identified that age is the only statistically significant predictor of a positive collagen impact on QoL (p=0.002), with younger individuals more likely to benefit from its usage. This higher efficacy in younger people may be due to better absorption and greater physiological responsiveness. While they are not statistically significant, higher educational level and monthly income showed near-significant trends, which suggests that sociodemographic factors may influence both usage and perceived effectiveness.

Limitations

There are several limitations of this study. The cross-sectional design prevents establishing a causal relationship and reflects only one time point. Self-reported data may be affected by recall and social desirability biases. Variation in collagen product formulations, dosages, and brands among participants reduces comparability. The short duration of supplement use may not capture the long-term effects. The lack of objective clinical measures also limits the confirmation of the subjective quality of life improvements. Finally, this sample is mainly from the Central Region, which may not be representative of all Saudi consumers.

Implications and future direction

Oral collagen supplementation can yield modest improvements in skin, hair, joint comfort, and sleep, which provide evidence-based information to clinicians and consumers about the realistic benefits and optimal usage durations. Future research should prioritize randomized controlled trials to confirm causality, explore dose-response relationships, and assess long-term safety across diverse populations. Furthermore, investigations into mechanisms that underlie systemic effects and potential adverse reactions are warranted. Additionally, there must be educational interventions to improve consumer knowledge and adherence, which could enhance outcomes and minimize premature discontinuation, and effective regulatory oversight.

## Conclusions

This study shows that oral collagen supplementation has a potential benefit on various aspects of quality of life, which include hair, skin, joint comfort, and sleep among Saudi consumers. The participants who used collagen supplements for longer durations have reported more favorable outcomes, while younger individuals were more likely to experience improvements. Despite these positive effects, there are some side effects, such as gastrointestinal discomfort and kidney issues, due to which early discontinuation of collagen supplements was common. Awareness regarding the appropriate use and potential outcomes was generally low.

## Appendices

Table 7 shows the survey questionnaire used in the present study.

**Table 7:** Collagen use survey

Number	Question
1	Sex
2	Age
3	Educational level
4	Monthly salary
5	Region of Saudi Arabia
6	Did you use any collagen formula before?
7	Choose the reasons for not using collagen
8	The extent of your knowledge about collagen
9	The reason for current use
10	Did you reach the expected result after a month of use on hair and nails?
11	Did you reach the expected result after a month of use on the skin?
12	Did you reach the expected result after a month of use on joints?
13	Did you reach the expected result after a month of use on protein levels?
14	Did you reach the expected result after a month of use on sleep improvement?
15	Number of sleeping hours before collagen
16	Number of sleeping hours after collagen
17	Rate the efficacy of collagen for healthy joints (out of 5)
18	Daily dose of collagen
19	How long did you continue using collagen?
20	Awareness of available collagen dosage forms
21	Most commonly used collagen dosage form
22	Awareness about collagen side effects
23	Most common side effects experienced
24	Did side effects lead to discontinuation of collagen?
25	Did you stop taking collagen before seeing results?
26	If yes, what was the reason for stopping?
